# Circular Economy in Membrane Technology

**DOI:** 10.3390/membranes13090784

**Published:** 2023-09-08

**Authors:** Junkal Landaburu-Aguirre, Serena Molina

**Affiliations:** IMDEA Water Institute, Punto Com. nº 2, 28805 Alcalá de Henares, Spain; serena.molina@imdea.org

The European Union (EU) produces more than 2.5 billion tonnes of waste every year. In this context, the EU is currently updating its legislation on waste management, in order to promote a shift to a more sustainable model known as the circular economy. The main objective of the circular economy is to keep the value of the materials and energy used in products for as long as possible, minimizing waste and the use of resources. For this purpose, actions must be taken at all stages of the life cycle of the product, from the extraction of the raw materials, through the material and product design, to waste management and recycling.

This Special Issue of *Membranes*, entitled “Circular economy in membrane technology”, aims to address membrane technology from a circular economy approach. In this way, it is focused on recent progress in membrane process developments, both at industrial and scientific levels, aiming to keep the membranes as long as possible within the value chain of their processes. Therefore, in this Special Issue, original research articles and reviews on fouling phenomena and fouling mitigation, as well as studies on alternative membrane management routes, such as membrane reuse and recycling, were welcomed.

In this Special Issue, Lejarazu-Larrañaga et al. [1] published a review article focused on the implementation of circular economy principles in RO technology through a comprehensive analysis of the RO membrane life cycle from the membrane manufacturing stage, through the usage of the membrane, to the end-of-life (EoL) membrane management. Within the membrane manufacturing stage, the authors emphasize the need for eco-designed RO modules by the incorporation of more sustainable components, such as biopolymers, recycled materials, and green solvents during the process. Further, regarding the implementation stage of membranes, the authors reviewed research papers focused on fouling prevention and mitigation by feed pre-treatment technologies, early fouling detection methods, and membrane cleaning protocols, which allow for improving the life span of the membranes. Regarding the EoL management of RO membranes, Lejarazu-Larrañaga et al. [1] analyze current membrane management patterns and review recent advances in EoL membrane reuse, recycling, and energy recovering alternatives, giving special attention to emerging indirect recycling strategies, as well as to the potential applications of Life Cycle Assessment (LCA) as a tool to evaluate the environmental impact associated with EoL membrane management.

This Special Issue also contains three research papers focused on the reuse and recycling of EoL RO membranes. The research paper of Garcia-Pacheco et al. [2] focuses on the implementation of EoL RO membranes and regenerated RO membranes for the treatment of landfill leachate. The study was conducted first at laboratory scale, then scaled up and operated during 27 months as a long-term test in a landfill leachate full-scale facility. The results of this study show that the studied membranes had a successful performance, obtaining good water quality during the treatment, which fits the required standards, and a trend of lower energy requirements.

The research papers of Senán-Salinas et al. [3] and Pompa-Pernía et al. [4] focused on direct and indirect recycling, respectively. Direct recycling is defined as the transformation of EoL RO membranes to UF and NF membranes, maintaining the original module design (in most of the cases, the spiral wound configuration), whilst in indirect recycling, an EoL RO membrane is disassembled, enabling the individual management and recycling of flat sheet membranes and other module components [1]. In this context, Senán-Salinas et al. [3] studied the recyclability and technological niches for converting EoL RO modules into nanofiltration (NF) membranes, using the Life Cycle Assessment (LCA) as an indicator of the global warming potential or carbon footprint. Particularly, the study focuses on defining which permeabilities and lifespans of the recycled NF membranes limit their implementation in water treatment, and under which operational conditions (i.e., feed pressure) and designs (i.e., the number of consecutive modules in a stage) the recycled NF membranes have advantageous outcomes in environmental terms.

Pompa-Pernía et al. [4] studied the preparation of NF and anion exchange membranes (AEM) to further implement them for the treatment of saline urban wastewater by NF and electrodialysis (ED). The prepared recycled membranes showed successful performance. For instance, recycled NF membranes obtained high selective rejection of divalent ions. Further, prepared recycled AEMs showed a suitable demineralization rate for the irrigation of crops, without compromising the power consumption. Therefore, the authors of this study concluded that the recycled membranes tested showed adequate potential in wastewater treatment for crop irrigation purposes, in terms of conductivity and SAR value.

Since the circular economy promotes the valorization of waste streams into products with high added value in the market, this Special Issue also focuses on the implementation of membrane processes for the recovery of valuable compounds. In this context, this Special Issue contains two research articles focused on the recovery of valuable compounds from biorefinery process streams. On the one hand, Rathnayake et al. [5] studied the valorization of hemicellulose (HMC) fraction obtained during the biorefinery processing of a lignocellulosic raw material using nanofiltration membranes. HMC is a diverse group of polysaccharides, which consists of various monosaccharide units such as glucose and xylose, valuable byproducts such as furfural and hydroxymethyl furfural, as well as impurities such as acetic acid, formic acid, and inorganic residues [6]. Separation of HMC sugars from byproducts and impurities is one of the major challenges in upstream processing in biorefineries. For the efficient upstream processing of HMC streams, one of the key strategies is developing membrane technologies to remove soluble non-sugar components and, concurrently, to achieve higher concentrations and ensure a higher purity of HMC sugars [7]. Rathnayake et al. [5] showed that using nanofiltration membranes, and lower pH and temperature, maximizes the monosaccharide and furfural retention, obtaining a successful separation from impurities such as acetic acid.

Further, Ribeiro et al. [8] focused on the *Nannochloropsis* sp. microalgae biorefinery process where, in addition to the enriched in lipids fraction, a soluble protein fraction can be produced for food applications, free from insoluble proteins and, ideally, without the presence of lipids and colored chlorophyll-a. Therefore, the aim of the work of Ribeiro et al. [8] was to define a suitable methodology for using membrane processing to obtain a high level of permeate flux with maximum soluble protein recovery (free from chlorophyll-a, insoluble proteins, and lipids). As they observed, the ultrafiltration process implemented obtained an enriched protein fraction, free from potentially undesirable contaminants. Further, they concluded that combination of ultrafiltration in a diafiltration mode, followed by concentration under controlled permeate flux conditions, enhances the soluble protein recovery in the permeate and allows a higher value of volumetric flux to be maintained for the entire diafiltration process.

In general, this Special Issue has collected important advances that tackle the circular economy approach in membrane technology from different perspectives. Therefore, the editors would like to thank the authors and reviewers for their valuable contributions to this Special Issue.
